# Dual Foot-Mounted Localisation Scheme Employing a Minimum-Distance-Constraint Kalman Filter Under Coloured Measurement Noise

**DOI:** 10.3390/mi15111346

**Published:** 2024-10-31

**Authors:** Yuan Xu, Jingwen Yu, Xiangpeng Wang, Teng Li, Mingxu Sun

**Affiliations:** School of Electrical Engineering, University of Jinan, Jinan 250022, China; cse_xuy@ujn.edu.cn (Y.X.); 202321201001@stu.ujn.edu.cn (J.Y.);

**Keywords:** minimum-distance-constraint, Kalman filter, coloured measurement noise (CMN)

## Abstract

This study proposes a dual foot-mounted localisation scheme with a minimum-distance-constraint (MDC) Kalman filter (KF) for human localisation under coloured measurement noise (CMN). The dual foot-mounted localisation employs inertial measurement unit (IMUs), one on each foot, and is intended for human navigation. The KF under CMN (cKF) is then derived from the data-fusion model of the proposed navigation scheme. Finally, the MDC condition is designed and an MDC–cKF model is proposed to reduce the error in the IMUs. Empirical results showed that the proposed method effectively improves the navigation accuracy from that of MDC–KF, which neglects the effect of CMN.

## 1. Introduction

As technology advances and human living standards improve [1], an increasing number of applications require the precise pose and posture information of the human body [2,3]. For instance, the authors of [4] determined the precise self-locations of humans using inertial measurement units (IMUs), which have delivered excellent results in human activity recognition [5]. The authors of [6] combined a microelectronic mechanical system with an inertial navigation system (INS) to capture the postures of human upper limbs. The combined system improves the rehabilitation training level of patients with motor function impairment. A pedestrian positioning system has been designed with inertial sensors under geometric constraints of the foot [7].

The mainstream methods of human positioning technology are INS [8], vision [8], and global navigation satellite systems (GNSSs) [9]. In these technologies, human localisation is often combined with vision tools. One of many approaches is visual-aided two-dimensional pedestrian navigation with a smartphone [10], which measures the heading-change information from consecutive images acquired by a visually aided sensor. The authors of [11] developed a two-dimensional indoor pedestrian navigation system that integrates the measurements of self-contained sensors. However, traditional methods cannot usually capture the complex scenarios of human body movements with sufficient accuracy. To enhance the accuracy of navigation, researchers have built virtual IMUs and combined them with gait-assisted pedestrian navigation methods. Note that the visual method requires high-quality images, which can be difficult to obtain. Moreover, the low speed of the visual method is unsuitable for some highly dynamic situations. Nevertheless, several visual-based methods have been proposed for human posture measurement [12]; for example, the authors of [13] applied an optical motion-capture system to the detection and classification of distortions. Their system employs derivative analysis, low-pass filtering, mathematical morphology, and a loose predictor. Its effectiveness was experimentally confirmed. Many systems based on GNSS-based human navigation have also been proposed. For instance, the global positioning system (GPS) navigation system of [14] fuses a dual-rate Kalman filter (KF) with GPS pseudoranges and INS measurements. The authors of [15] designed a GPS/INS integrated navigation system that achieves seamless navigation. Although GNSS-based navigation methods can stably perform in outdoor environments, interference of GNSS signals largely reduces their positioning accuracy in indoor environments. Other researchers have focussed on INS-based human navigation. For instance, the authors of [16] designed an improved step-length estimator for pedestrian dead reckoning (PDR), and the authors of [17] proposed a foot-mounted PDR employing a particle filter with an adaptive-weight updating strategy.

The INS-based methods achieve self-navigation but tend to accumulate errors. Some short-distance communication technologies can obtain the position of a target object when GNSS measurements are unavailable. For instance, an ultra-wide band can assist the INS in indoor localisation [18]. The authors of [19] proposed a global localisation method using a radio frequency identification (RFID)-based mobile robot, which combines two types of RFID signal information. An accurate WiFi-based localisation for smartphones [20] and visible light positioning that improves the indoor localisation accuracy [21] have also been proposed. In the latter scheme, noise is measured from the Allan variance and mitigated with adaptive least squares and extended KF (EKF) positioning algorithms. However, localisation technologies require reference equipment that must be properly placed to ensure accurate localisation. In summary, the existing technologies have individual strengths and limitations. Visual localisation technologies require high image quality and their processing speed is slow. GNSS is provide stable solutions but are prone to outage in indoor environments. Although short-communication technologies can locate a target human in indoor environments, they require reference equipment. INS-based methods require no reference equipment but tend to accumulate errors. Noted that the advantages of the method using only INS without GPS is its high sampling rate, moreover, the INS-based solution is one seamless solution, since the sensor of the INS can continuously output data.

Considering its comparative advantage, INS-based human navigation is adopted in the present study. The localisation accuracy can be further improved by localisation-system-based data-fusion methods. KFs have been widely used in such systems [22,23]. For instance, the authors of [8] proposed an event-triggered multi-rate size-variable KF for an outdoor navigation system. The authors of [24] combined a magnetic-field-gradient-based EKF with a bidirectional long short-term memory network for estimating the velocities of moving objects. Employing a robust KF based on the *Mahalanobis* distance, the authors of [25] resolved the interference among a strap-down inertial navigation system, Doppler velocity log, and an ultrashort baseline system in a complex underwater environment. The results validated the velocity estimation and possible positions in different sensor-deployment and trajectory scenarios. The authors of [21] proposed an optimised two-filter filter smoothing technology with a KF ensemble, which provides cost-effective and accurate localisation. They applied their technology to indoor mobile robots in the Internet of Things environment. In [26], the localisation accuracy was improved by a KF-based model that estimates the covariance of process noise. However, the above-mentioned KF methods do not consider the impact of coloured measurement noise (CMN), in [3], the dual KF under the CMN is consider, which is effective to reduce the influence of CMN to the INS-based integrated human localisation. Applying the backward Euler (BE) method, the authors of [27] proposed a KF under CMN that well fits non-feedback systems. Meanwhile, although the Zero Velocity Update (ZUPT) method can reduce the error accumulation of the INS, it also need other methods to limit errors, and the existing INS-based methods impose no physical constraints during the human walking process.

Although CMN will interfere with localisation processing, thus affecting the accuracy of human localisation, it has been neglected in the existing approaches. Note that although the GNSS-based localisation methods have been used widely, there are some specific applications; for example, in firefighting, the workers work in a sealed environment, and so on. It should be pointed out that the potential application should be in the GNSS outage area, and the short communication-technologies-based localisation method is not available. In these specific applications, the accuracy of these methods may significantly decrease, and the advantage of the INS’s strong autonomy is particularly suitable for these scenarios. However, it should be pointed out that in these scenarios, the CMN will affect the accuracy of the localisation. Thus, to achieve human localisation under CMN, we propose a dual foot-mounted IMU-based localisation scheme employing a KF under minimum distance constraints (MDCs). This human navigation scheme measures the positions of the right and left feet with two IMUs, one mounted on each foot. The measured positions are input to a data-fusion filter that outputs the position of the target person. Based on the data-fusion model of the human navigation scheme, we then derive a KF under CMN (cKF). Finally, we design the MDC condition and propose an MDC–cKF that reduces the error in the IMUs. Judging from the empirical results, the proposed method effectively improves the accuracy of human navigation.

The achievements of our study are summarised below:

- We first design a human-localisation scheme employing dual foot-mounted IMUs. The foot-mounted IMUs are individually affixed to the left and right feet, one IMU on each foot. The two IMUs measure the acceleration, gyroscopic, and magnetometer data in parallel. The obtained data of the left or right foot are input to a data-fusion filter that outputs the position of the corresponding foot.
- Based on the dual foot-mounted IMU-based human localisation scheme, we derive the cKF. In this derivation, we employ the colour factor and modify the traditional KF using the BE method to reduce the CMN. The modified KF is operated during the stance stage of the human foot.
- Third, we propose a KF under CMN constraints. Here, we impose the MDC condition because a minimum distance between the feet on the plane was observed during human walking. When constrained by this point, the KF effectively reduced the INS’s position error of the left or right foot.
- Through experimental evaluations, we confirm that the presented algorithms far outperform their conventional counterparts. Practical tests of the two IMUs for human localisation, coupled with real-time-kinematic (R-T-K)-provided reference values, demonstrate that the proposed cKF is notably more effective than the traditional KF.

The remaining sections of this paper are organised as follows. Section 2 introduces the dual foot-mounted IMU-based localisation scheme and formulates the problem. Section 3 describes the improved MDC–cKF, and Section 4 experimentally assesses the positioning accuracy of MDC–KF. The paper concludes with Section 5.

## 2. Design and Formulation of the Dual Foot-Mounted IMU-Based Localisation Scheme

In this section, we design the dual foot-mounted IMU-based localisation scheme and formulate the corresponding problem.

### 2.1. Dual Foot-Mounted IMU-Based Localisation Scheme

**Figure 1:**
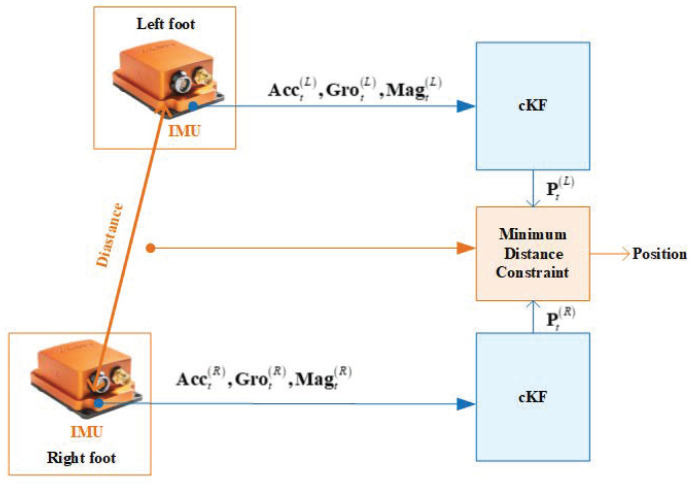
Structure of the dual foot-mounted IMU-based localisation scheme with a minimum distance-constraint Kalman filter under coloured measurement noise.

We first introduce the dual foot-mounted IMU-based localisation scheme. As shown in Figure 1, the localisation scheme uses two foot-mounted IMUs, one fixed on the right foot, the other on the left foot. The two IMUs measure the acceleration ${\mathrm{Acc}}_{t}$, gyroscopic data ${\mathrm{Gro}}_{t}$, and magnetometer data ${\mathrm{Mag}}_{t}$ of each foot in parallel. These data are input to the cKF, which will be described in the following subsection. The variables are superscripted by (L) and (R) denoting the left and right feet, respectively. Meanwhile, we assume a minimum distance between the left and right feet to structurally constrain $P_{t}^{\left(L\right)}$ and $P_{t}^{\left(R\right)}$. Finally, the position is outputted.

### 2.2. Problem Formulation

The state equation of the cKF on the left foot is given by

(1) $$\underset{x_{l}^{\left(L\right)-}}{\underset{︸}{\left[\begin{array}{c} \phi_{l}^{\left(L\right)-}\\ \delta V_{l}^{\left(L\right)-}\\ \delta P_{l}^{\left(L\right)-}\\ b_{l}^{\left(L\right)-}\\ \varepsilon_{l}^{\left(L\right)-} \end{array}\right]}}=\underset{T_{l}^{\left(L\right)}}{\underset{︸}{\left[\begin{array}{ccccc} I_{3\times 3} & 0_{3\times 3} & 0_{3\times 3} & 0_{3\times 3} & -R_{b}^{n}\Delta l\\ S\left(a_{l}^{\left(n\right)}\right)\Delta l & I_{3\times 3} & 0_{3\times 3} & R_{b}^{n}\Delta & 0_{3\times 3}\\ 0_{3\times 3} & \Delta lI_{3\times 3} & I_{3\times 3} & 0_{3\times 3} & 0_{3\times 3}\\ 0_{3\times 3} & 0_{3\times 3} & 0_{3\times 3} & I_{3\times 3} & 0_{3\times 3}\\ 0_{3\times 3} & 0_{3\times 3} & 0_{3\times 3} & 0_{3\times 3} & I_{3\times 3} \end{array}\right]}}\underset{x_{l}^{\left(L\right)}}{\underset{︸}{\left[\begin{array}{c} \phi_{l}^{\left(L\right)}\\ \delta V_{l}^{\left(L\right)}\\ \delta P_{l}^{\left(L\right)}\\ b_{l}^{\left(L\right)}\\ \varepsilon_{l}^{\left(L\right)} \end{array}\right]}}+w_{t}^{\left(L\right)}\;,$$

(2) $$S\left(a_{t}^{\left(n\right)}\right)=\left[\begin{array}{ccc} 0 & a_{Ut}^{\left(n\right)} & -a_{Nt}^{\left(n\right)}\\ -a_{Ut}^{\left(n\right)} & 0 & a_{Et}^{\left(n\right)}\\ a_{Nt}^{\left(n\right)} & -a_{Et}^{\left(n\right)} & 0 \end{array}\right]\;,$$

where Δt is the sample time, $x_{t}^{\left(L\right)}={\left[\begin{array}{ccccc} \phi_{t}^{\left(L\right)} & \delta V_{t}^{\left(L\right)} & \delta P_{l}^{\left(L\right)} & b_{t}^{\left(L\right)} & \varepsilon_{t}^{\left(L\right)} \end{array}\right]}^{T}$ is the state vector. Here, $\phi_{t}^{\left(L\right)}$ is the attitude error, $\delta V_{t}^{\left(L\right)}$ is the velocity error in the East–North–Up frame (n-frame), $b_{t}^{\left(L\right)}$ is the acceleration error in the n-frame, $\varepsilon_{t}^{\left(L\right)}$ is the gyroscope error in the body-frame (b-frame), and $w_{t}^{\left(L\right)}$ is the system noise with covariance $Q_{t}^{\left(L\right)}$.

Whether the foot is in a landing state is determined from the value of the accelerometer $V_{{\mathrm{Acc}}_{t}^{\left(L\right)}}$, formulated as follows:

(3) $$V_{{\mathrm{Acc}}_{t}^{\left(L\right)}}={\left({\left({\mathrm{Acc}}_{x,t}^{\left(L\right)}\right)}^{2}+{\left({\mathrm{Acc}}_{y,t}^{\left(L\right)}\right)}^{2}+{\left({\mathrm{Acc}}_{z,t}^{\left(L\right)}\right)}^{2}\right)}^{\frac{1}{2}}\;,$$

where $\left({\mathrm{Acc}}_{x,t}^{\left(L\right)},{\mathrm{Acc}}_{y,t}^{\left(L\right)},{\mathrm{Acc}}_{z,t}^{\left(L\right)}\right)$ denote the value of the left foot’s accelerometer in the b-frame. We first set a threshold t_d such that if $V_{{\mathrm{Acc}}_{t}^{\left(L\right)}}\leq t\_d$, the left foot is on the floor. In this case, the cKF filter is activated and performs the following measurement:

(4) $$\underset{m_{t}^{\left(L\right)-}}{\underset{︸}{\left[{\hat{V}}_{t}^{\left(L\right)}-0\right]}}=\underset{H}{\underset{︸}{\left[\begin{array}{ccccc} 0_{3\times 3} & I_{3\times 3} & 0_{3\times 3} & 0_{3\times 3} & 0_{3\times 3} \end{array}\right]}}x_{t}^{\left(L\right)-}+\underset{{\bar{v}}_{t}^{\left(L\right)}}{\underset{︸}{\alpha {\bar{v}}_{t-1}^{\left(L\right)}+v_{t}^{\left(L\right)}}}\;,$$

where ${\hat{V}}_{t}^{\left(L\right)}$ is the value of $V_{t}^{\left(L\right)}$ measured by the IMU on the left foot at time index *t*. When the foot is on the floor, the reference velocity of the INS is zero, so $\left[{\hat{V}}_{t}^{\left(L\right)}-0\right]$ is the measurement vector $m_{t}^{\left(L\right)}$. The terms ${\bar{v}}_{t}^{\left(L\right)}$ and ${\bar{v}}_{t-1}^{\left(L\right)}$ are the CMN at time indices *t* and t−1, respectively, $v_{t}^{\left(L\right)}∼N\left(0,R_{t}^{\left(L\right)}\right)$ is Gaussian noise with covariance $R_{t}^{\left(L\right)}$, and α is the colour factor. Note that after replacing the superscript (L) with (R) in Equation (3), we obtain the model of the right foot.

## 3. Minimum-Distance-Constraint Kalman Filter Under Coloured Measurement Noise

This subsection describes the designed cKF under CMN. We first derive the cKF from the model given by Equations (1)–(4). We then describe the MDC method that assists the cKF.

### 3.1. Kalman Filter Under Coloured Measurement Noise

Here, we derive the cKF based on Equations (1)–(4). To this end, we rewrite Equation (4) as follows:

(5) $$\begin{array}{ccc} {\bar{m}}_{t}^{\left(L\right)} & = & m_{t}^{\left(L\right)}-\alpha m_{t-1}^{\left(L\right)}\\ & = & {\mathrm{Hx}}_{t}^{\left(L\right)-}+{\bar{v}}_{t}^{\left(L\right)}-\alpha {\mathrm{Hx}}_{t-1}^{\left(L\right)-}-\alpha {\bar{v}}_{t-1}^{\left(L\right)}\;, \end{array}$$

Taking $x_{t}^{(L)-}$ and the ${\bar{v}}_{t-1}^{\left(L\right)}$ in Equation (5) and applying Equation (1), we obtain

(6) $$\begin{array}{c} {\bar{m}}_{t}^{\left(L\right)}={\bar{H}}_{t}^{\left(L\right)}x_{t}^{\left(L\right)-}+{\bar{\bar{v}}}_{t}^{\left(L\right)}\;, \end{array}$$

where

(7) $${\bar{H}}_{t}^{\left(L\right)}=H-{\bar{T}}_{t}\;,$$

(8) $${\bar{T}}_{t}=\alpha H{\left(T_{l}^{\left(L\right)}\right)}^{-1}\;,$$

(9) $${\bar{\bar{v}}}_{t}^{\left(L\right)}={\bar{T}}_{t}w_{t}^{\left(L\right)}+v_{t}^{\left(L\right)}\;,$$

The covariance ${\bar{R}}_{t}^{\left(L\right)}$ of the ${\bar{\bar{v}}}_{t}^{\left(L\right)}$ is calculated as

(10) $${\bar{R}}_{t}^{\left(L\right)}=E\left({\bar{\bar{v}}}_{t}^{\left(L\right)}{\left({\bar{\bar{v}}}_{t}^{\left(L\right)}\right)}^{T}\right)={\bar{T}}_{t}\theta_{t}+R_{t}^{\left(L\right)}\;,$$

where $\theta_{t}=Q_{t}^{\left(L\right)}{\left({\bar{T}}_{t}\right)}^{T}$. The prior error covariance to the KF can be computed as

(11) $$P_{t}^{\left(L\right)-}=T_{l}^{\left(L\right)}P_{t-1}^{\left(L\right)}{\left(T_{l}^{\left(L\right)}\right)}^{T}+Q_{l}^{\left(L\right)}\;,$$

From Equation (6), we can obtain the measurement residual as follows:

(12) $$\begin{array}{ccc} {\mathrm{mr}}_{t}^{\left(L\right)} & = & {\bar{m}}_{t}^{\left(L\right)}-{\bar{H}}_{t}^{\left(L\right)}{\hat{x}}_{t}^{\left(L\right)-}\\ & = & {\bar{H}}_{t}^{\left(L\right)}x_{t}^{\left(L\right)}+{\bar{\bar{v}}}_{t}^{\left(L\right)}-{\bar{H}}_{t}^{\left(L\right)}{F\hat{x}}_{t-1}^{\left(L\right)}\\ & = & {\bar{H}}_{t}^{\left(L\right)}F\underset{\eta_{t}^{\left(L\right)}}{\underset{︸}{\left(x_{t}^{\left(L\right)}-{\hat{x}}_{t}^{\left(L\right)}\right)}}+{\bar{H}}_{t}^{\left(L\right)}w_{t}^{\left(L\right)}+{\bar{\bar{v}}}_{t}^{\left(L\right)}\;, \end{array}$$

${\mathrm{Mr}}_{t}^{\left(L\right)}=E\left({\mathrm{mr}}_{t}^{\left(L\right)}{\left({\mathrm{mr}}_{t}^{\left(L\right)}\right)}^{T}\right)$ is then obtained as

(13) $$\begin{array}{ccc} {\mathrm{Mr}}_{t}^{\left(L\right)} & = & {\bar{H}}_{t}^{\left(L\right)}{\mathrm{FP}}_{t-1}^{\left(L\right)}F^{T}{\left({\bar{H}}_{t}^{\left(L\right)}\right)}^{T}+{\bar{H}}_{t}^{\left(L\right)}Q_{t}^{\left(L\right)}{\left({\bar{H}}_{t}^{\left(L\right)}\right)}^{T}+{\bar{T}}_{t}\theta_{t}+R_{t}^{\left(L\right)}+{\bar{H}}_{t}^{\left(L\right)}\theta_{t}+\theta_{t}^{T}{\left({\bar{H}}_{t}^{\left(L\right)}\right)}^{T}\\ & = & {\bar{H}}_{t}^{\left(L\right)}P_{t}^{\left(L\right)-}{\left({\bar{H}}_{t}^{\left(L\right)}\right)}^{T}+R_{t}^{\left(L\right)}+H_{t}^{\left(L\right)}\theta_{t}+\theta_{t}^{T}{\left({\bar{H}}_{t}^{\left(L\right)}\right)}^{T}\;, \end{array}$$

Thus,

(14) $$\begin{array}{ccc} {\hat{x}}_{t}^{\left(L\right)} & = & {\hat{x}}_{t}^{\left(L\right)-}+K_{t}^{\left(L\right)}{\mathrm{mr}}_{t}^{\left(L\right)}\\ & = & {F\hat{x}}_{t-1}^{\left(L\right)}+K_{t}^{\left(L\right)}\left({\bar{m}}_{t}^{\left(L\right)}-{\bar{H}}_{t}^{\left(L\right)}{\hat{x}}_{t-1}^{\left(L\right)}\right)\;, \end{array}$$

In this work, we insert a new term $\Lambda_{t}^{\left(L\right)}\left({\bar{m}}_{t}^{\left(L\right)}-{\bar{H}}_{t}^{\left(L\right)}x_{t}^{\left(L\right)-}-{\bar{\bar{v}}}_{t}^{\left(L\right)}\right)$ into Equation (1):

(15) $$\begin{array}{ccc} x_{t}^{\left(L\right)-} & = & T_{t}^{\left(L\right)}x_{t}^{\left(L\right)}+w_{t}^{\left(L\right)}+\Lambda_{t}^{\left(L\right)}\left({\bar{m}}_{t}^{\left(L\right)}-{\bar{H}}_{t}^{\left(L\right)}x_{t}^{\left(L\right)-}-{\bar{\bar{v}}}_{t}^{\left(L\right)}\right)\\ & = & A_{t}^{\left(L\right)}x_{t}^{\left(L\right)}+\mu_{t}^{\left(L\right)}+\gamma_{t}^{\left(L\right)}\;, \end{array}$$

where

(16) $$A_{t}^{\left(L\right)}=\left(I-\Lambda_{t}^{\left(L\right)}{\bar{H}}_{t}^{\left(L\right)}\right)F\;,$$

(17) $$\mu_{t}^{\left(L\right)}=\Lambda_{t}^{\left(L\right)}{\bar{m}}_{t}^{\left(L\right)}\;,$$

(18) $$\gamma_{t}^{\left(L\right)}=\left(I-\Lambda_{t}^{\left(L\right)}{\bar{H}}_{t}^{\left(L\right)}\right)w_{t}^{\left(L\right)}-\Lambda_{t}^{\left(L\right)}{\bar{\bar{v}}}_{t}^{\left(L\right)}\;,$$

The covariance $Q_{t}^{\left(L\right)}$ of the system noise $\gamma_{t}^{\left(L\right)}∼N\left(0,Q_{t}^{\left(L\right)}\right)$ can be written as follows:

(19) $$\begin{array}{ccc} Q_{t}^{\left(L\right)} & = & E\left(\gamma_{t}^{\left(L\right)}{\left(\gamma_{t}^{\left(L\right)}\right)}^{T}\right)\\ & = & E\left(\left(\left(I-\Lambda_{t}^{\left(L\right)}{\bar{H}}_{t}^{\left(L\right)}\right)w_{t}^{\left(L\right)}-\Lambda_{t}^{\left(L\right)}{\bar{\bar{v}}}_{t}^{\left(L\right)}\right){\left(\left(I-\Lambda_{t}^{\left(L\right)}{\bar{H}}_{t}^{\left(L\right)}\right)w_{t}^{\left(L\right)}-\Lambda_{t}^{\left(L\right)}{\bar{\bar{v}}}_{t}^{\left(L\right)}\right)}^{T}\right)\\ & = & \left(I-\Lambda_{t}^{\left(L\right)}H\right)Q_{t}^{\left(L\right)}{\left(I-\Lambda_{t}^{\left(L\right)}H\right)}^{T}+\Lambda_{t}^{\left(L\right)}R_{t}^{\left(L\right)}{\left(\Lambda_{t}^{\left(L\right)}\right)}^{T}\;, \end{array}$$

To ensure that $\gamma_{t}^{\left(L\right)}$ and ${\bar{\bar{v}}}_{t}^{\left(L\right)}$ are uncorrelated, we set $E\left(\gamma_{t}^{\left(L\right)}{\left({\bar{\bar{v}}}_{t}^{\left(L\right)}\right)}^{T}\right)=0$, thereby obtaining

(20) $$\Lambda_{t}^{\left(L\right)}=\theta_{t}^{T}{\left(H\theta_{t}^{T}+R_{t}^{\left(L\right)}\right)}^{-1}\;,$$

And

(21) $$Q_{t}^{\left(L\right)}=\left(I-\Lambda_{t}^{\left(L\right)}{\bar{H}}_{t}^{\left(L\right)}\right)Q_{t}^{\left(L\right)}{\left(I-\Lambda_{t}^{\left(L\right)}{\bar{H}}_{t}^{\left(L\right)}\right)}^{T}\;,$$

The cKF is implemented through Algorithm 1.
**Algorithm 1:** The cKF for on the left foot
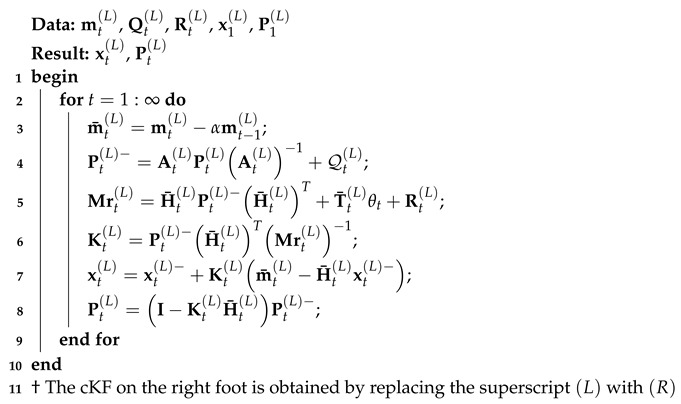


### 3.2. Minimum-Distance-Constraint Filter

In the above-mentioned cKF, the position of the left foot $r_{t}^{\left(L\right)}$ equals the position ${\hat{P}}_{t}^{\left(L\right)}$ measured by the IMU of the left foot minus the output $x_{t}^{\left(L\right)}$ of the cKF on the left foot. An identical calculation obtains the position $r_{t}^{\left(R\right)}$ of the right foot. The distance between the two feet of the walking target is computed as

(22) $$d_{w}=∥r_{t}^{\left(L\right)}-r_{t}^{\left(R\right)}∥\;,$$

Here, we extend the state vector to $r_{m,t}=\left[\begin{array}{c} r_{t}^{\left(L\right)}\\ r_{t}^{\left(R\right)} \end{array}\right]$ and determine the distance between the two feet in the east–north frame when at least one foot lands on the ground. This distance is set as the constraint vector $D_{m,t}$. The state estimation under the distance constraint in the dual-foot localisation system is then formulated as the following optimisation problem:

(23) $${\bar{r}}_{m,t}=\underset{{\overset{⌢}{r}}_{m,t}}{\arg\min}{\left({\overset{⌢}{r}}_{m,t}-r_{m,t}\right)}^{T}P_{m,t}^{-1}\left({\overset{⌢}{r}}_{m,t}-r_{m,t}\right),$$

(24) $$s.t.\;\underset{g\left(x_{m,t}\right)}{\underset{︸}{{∥P_{t}^{\left(L\right)}-P_{t}^{\left(R\right)}∥}^{2}}}-d_{c}^{2}=0\;,$$

where $P_{m,t}=\left[\begin{array}{cc} P_{t}^{\left(L\right)} & 0\\ 0 & P_{t}^{\left(R\right)} \end{array}\right]$, ${\bar{r}}_{m,t}$ is the constrained estimate of $r_{m,t}$, ${\overset{⌢}{r}}_{m,t}$ is an optimisation optimised variable of $r_{m,t}$ dc is the minimum distance. The final output of the filter is thus given by

(25) $$\begin{array}{ccc} {\bar{r}}_{m,t} & = & r_{m,t}-P_{m,t}^{-1}{\left(g^{\prime }\left({\bar{x}}_{m,t}\right)\right)}^{T}{\left(g^{\prime }\left({\bar{x}}_{m,t}\right)P_{m,t}^{-1}{\left(g^{\prime }\left({\bar{x}}_{m,t}\right)\right)}^{T}\right)}^{-1}\\ & & \left(g^{\prime }\left({\bar{x}}_{m,t}\right)x_{m,t}-d_{c}^{2}+g^{\prime }\left({\bar{x}}_{m,t}\right){\bar{x}}_{m,t}-g\left({\bar{x}}_{m,t}\right)\right)\;, \end{array}$$

## 4. Test

This section investigates the performance of the proposed method. We first describe the verification experiments. We then compare the localisation performance of the proposed MDC–KF under CMN with that of the MDC–KF.

### 4.1. Experimental Design

**Figure 2:**
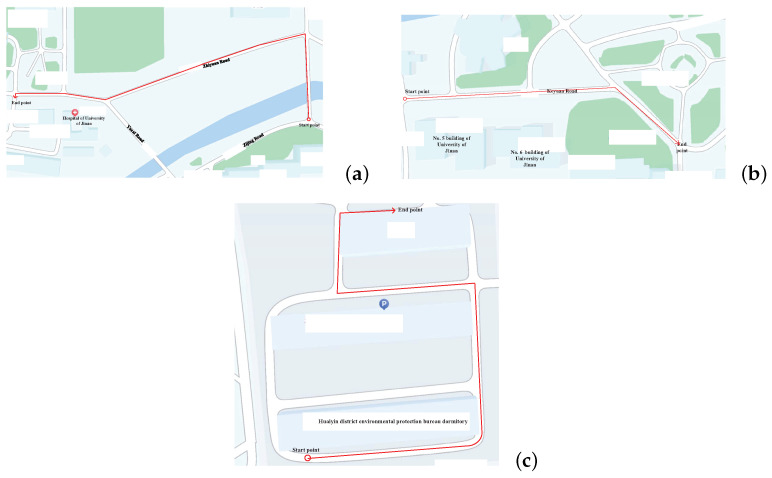
The planned paths used in this work of the present experiment: (**a**) planned path 1, (**b**) planned path 2, (**c**) planned path 3.

This subsection designs the verification experiments. The evaluation was performed at the University of Jinan, Shandong, China (see Figure 2 for the layout). The target human walked along two planned paths: one near the Hospital of the University of Jinan (approximate length 300 m), the other between Building No. 5 and Building No. 6 (approximate length 150 m). Moreover, we do the test again near the Huaiyin district environmental protection bureau dormitory in Jinan (approximate length 200 m).

**Figure 3:**
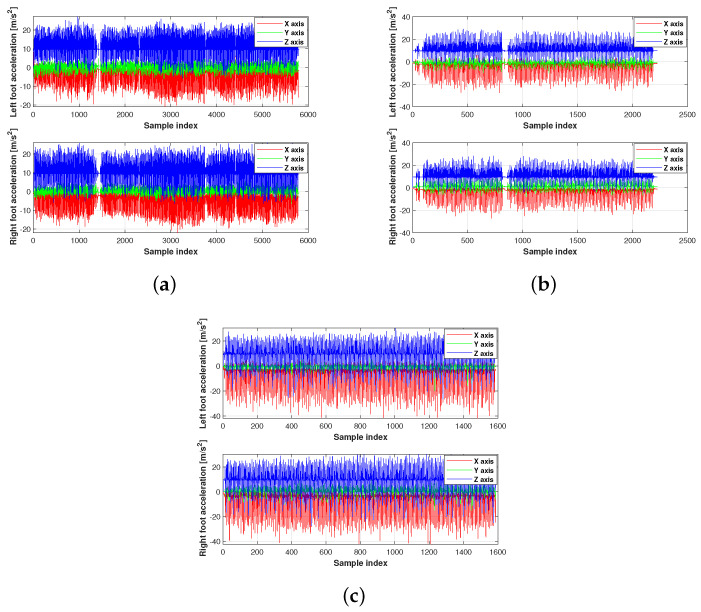
The acceleration value of the present experiment used in this work: (**a**) planned path 1, (**b**) planned path 2, (**c**) planned path 3.

**Figure 4:**
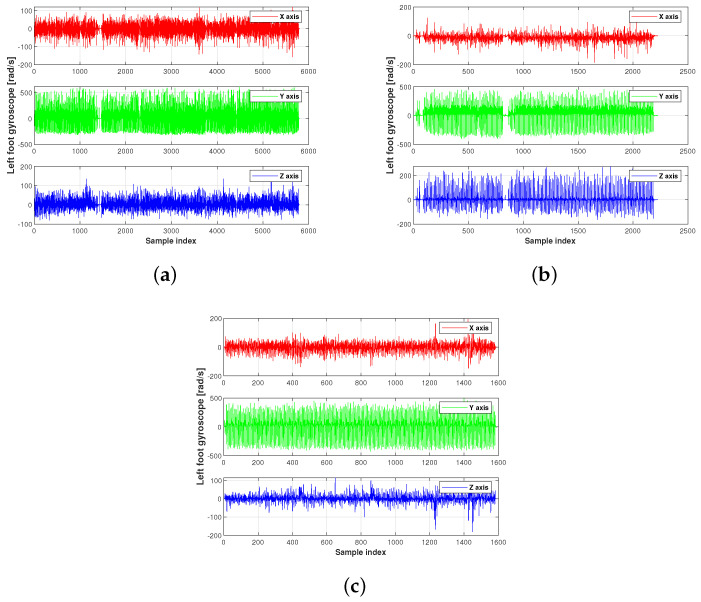
The left foot’s gyroscope value of the present experiment used in this work: (**a**) planned path 1, (**b**) planned path 2, (**c**) planned path 3.

**Figure 5:**
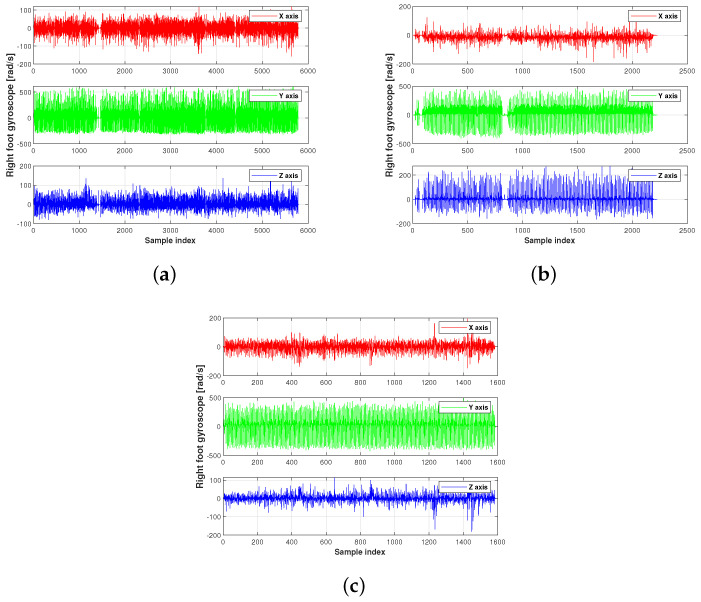
The right foot’s gyroscope value of the present experiment used in this work: (**a**) planned path 1, (**b**) planned path 2, (**c**) planned path 3.

**Figure 6:**
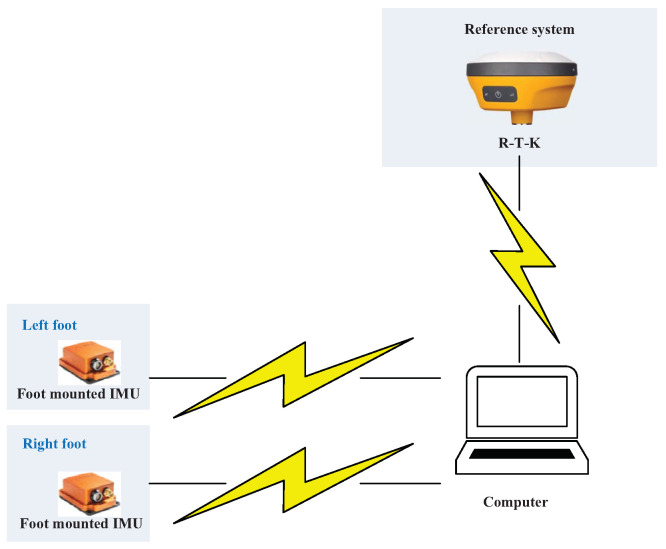
Structure of the testbed used in this work experiment.

**Figure 7:**
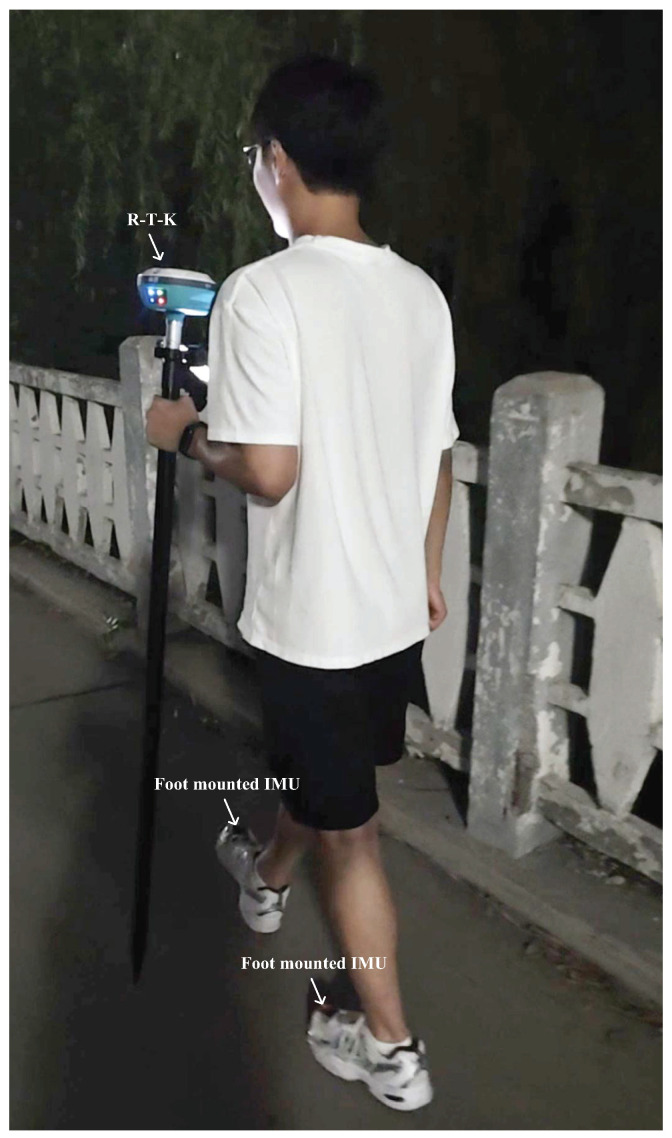
The target human used in this work subject of the present experiment.

The acceleration and gyroscope value of the present experiment used in this work are shown in Figure 3, Figure 4 and Figure 5. Figure 6 displays the testbed structure and the human subject wearing the sensors. The positions of the left and right feet sensors were measured by two Xsens DOT foot-mounted IMUs. We also obtained the reference value from an R-T-K sensor. The data of all sensors were sent to the computer via wireless communication. The human subject walked for approximately 200 m along the red line shown in Figure 7. Following [3,28], we also obtained the reference values from an R-T-K sensor. The essential parameters of the Xsens DOT IMUs and R-T-K are listed in Table 1 and Table 2, respectively.

### 4.2. Localisation Performance Along Planned Path 1

**Figure 8:**
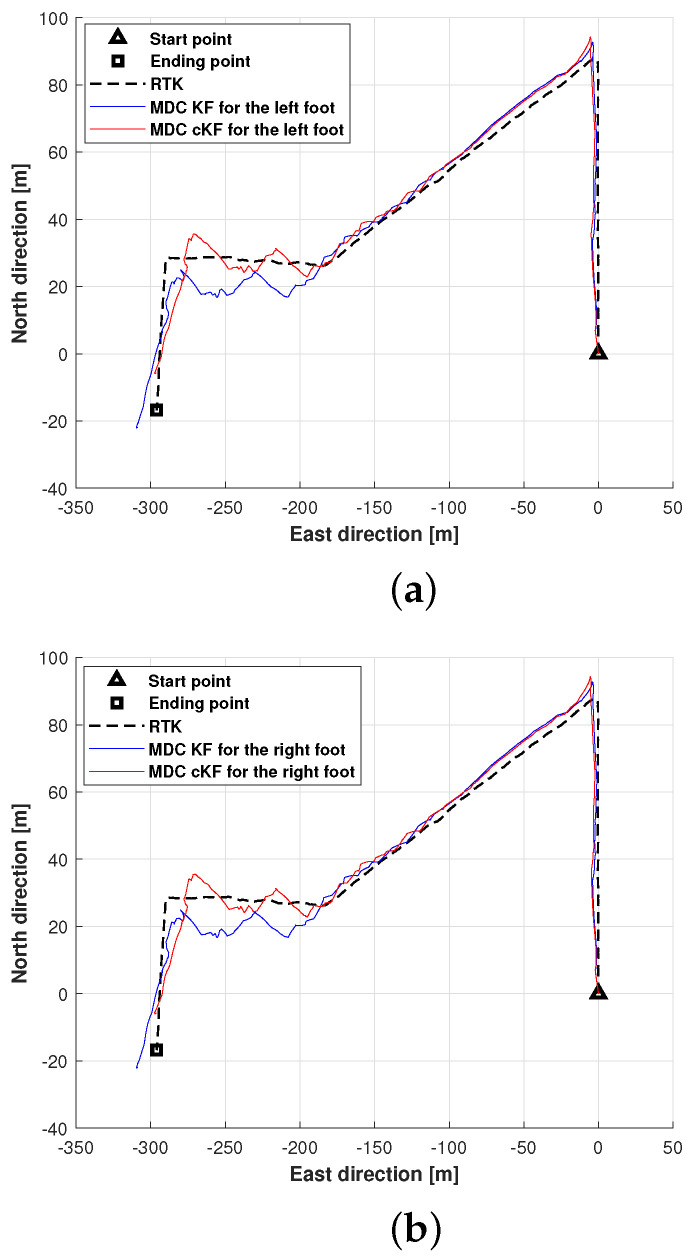
Foot positions along planned path 1 (black dashed lines) measured by the MDC–KF and MDC–cKF methods (solid blue and red lines, respectively): (**a**) left foot, (**b**) right foot.

This subsection evaluates and discusses the localisation performance of the proposed method along planned path 1. Here, the MDC–KF method was employed as the benchmark. The planned path and the positions of the left and right feet measured by MDC–KF and MDC–cKF are shown in Figure 8. Although the filter effectively reduced the localisation error, errors accumulated in the results of both methods (Figure 8a). The left-foot position estimated by MDC–cKF more closely approached the planned path than that estimated by MDC–KF. A similar result was obtained for the right foot (Figure 8b). Both filters reduced the localisation errors, but the localisation effect of the proposed method surpassed that of the MDC–KF method.

**Figure 9:**
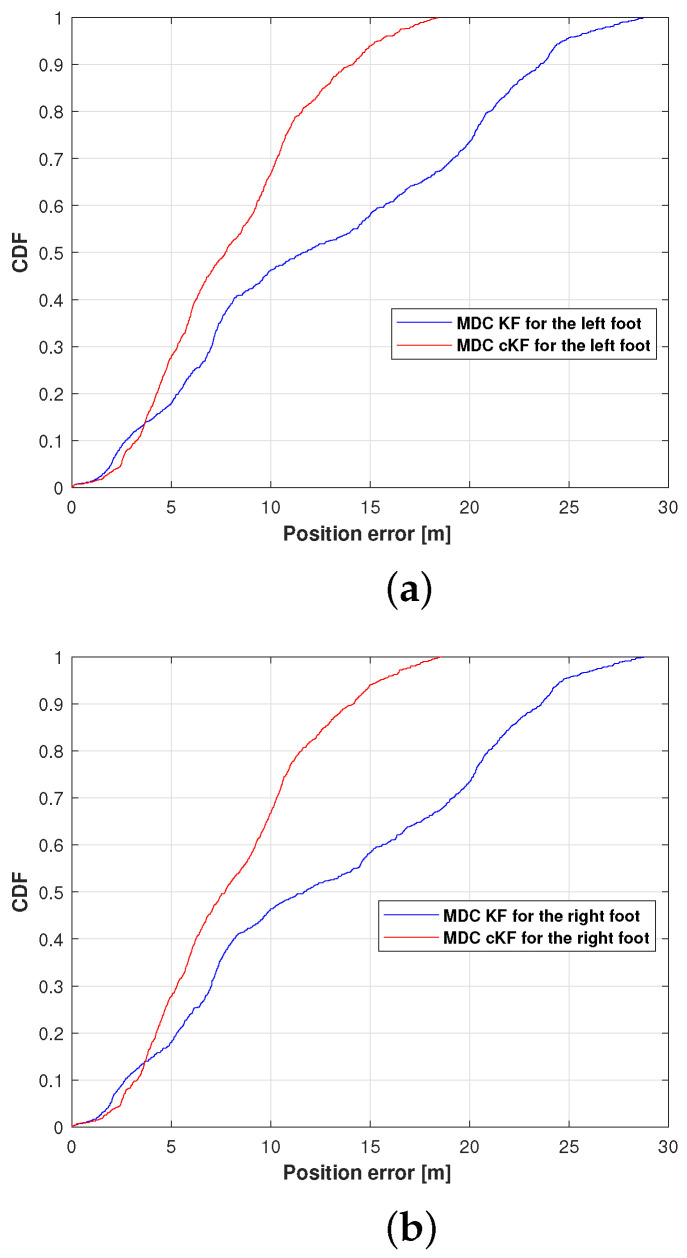
Position-error cumulative distribution functions (CDFs) of the MDC–KF and MDC–cKF methods (solid blue and solid red lines, respectively) along planned path 1: (**a**) left foot, (**b**) right foot.

Figure 9 shows the cumulative distribution functions (CDFs) of the left foot and right foot position errors in the MDC–KF and MDC–cKF methods. As presented in Figure 9a, the position error at the 0.9 point on the CDF axis was lower in the proposed MDC–cKF than in MDC–KF, confirming the effectiveness of the proposed method. Quantitatively, the proposed method reduced the position error at 0.9 CDF from 23.57 to 14.13 m, an improvement of 40.05%. The results of the right foot (Figure 9b) also demonstrate that the proposed method reduces the position error at the 0.9 CDF point. On this foot, the proposed method reduced the position error from 23.61 to 14.14 m, indicating a 40.11% reduction in localisation error.

**Figure 10:**
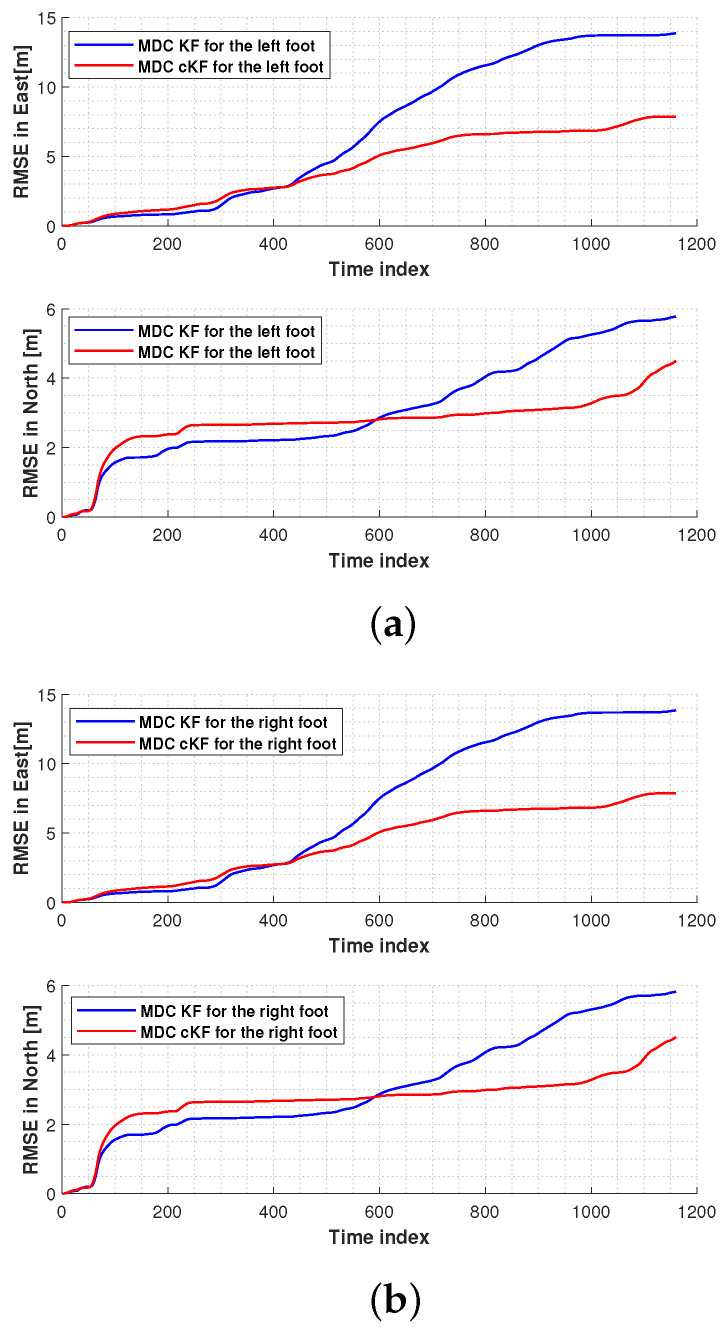
Root mean square errors (RMSEs) in the foot positions measured by the MDC–KF and MDC–cKF methods (solid blue and solid red lines, respectively) along planned path 1: (**a**) left foot, (**b**) right foot.

Figure 10 shows the root mean square errors (RMSEs) in the left-foot and right-foot positions measured by the MDC–KF and MDC–cKF methods. In both the east and north directions, the proposed MDC–cKF method more effectively reduced the localisation error of the left foot than the MDC–KF method (Figure 10a). A similar result was obtained for the right foot (Figure 10b).

To show the efficacy of the introduced methodology, we list the foot-position RMSEs of the MDC-KF and MDC–cKF methods in Table 3 and Table 4, respectively. The MDC–KF method yielded precise mean position errors of 9.8287 m and 9.8316 m for the left and right foot, respectively. The proposed MDC–cKF method reduced these errors to 6.1831 m and 6.1918 m, respectively, demonstrating improvements of 37.09% and 37.02%, respectively. All results demonstrate that the proposed method substantially improves the localisation accuracy from that of MDC–KF.

### 4.3. Localisation Performance Along Planned Path 2

**Figure 11:**
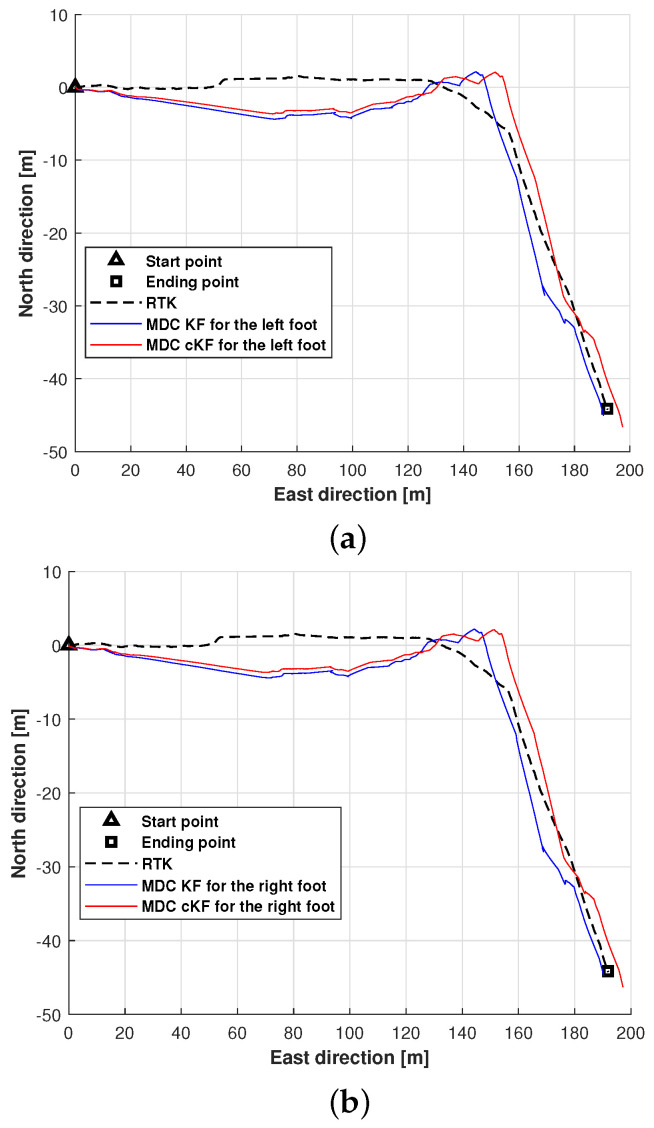
Foot positions along planned path 2 (black dashed lines) measured by the MDC–KF and MDC–cKF methods (solid blue and red lines, respectively): (**a**) left foot, (**b**) right foot.

This subsection evaluates and discusses the localisation performance of the proposed method along planned path 2, again employing MDC–KF as the benchmark method. The positions of the left and right feet measured by MDC–KF and MDC–cKF along planned path 2 are shown in Figure 11. Although the filters effectively reduced the localisation error, neither method prevented error accumulation (Figure 11a). The positions of both feet more closely approached the planned path when estimated by MDC–cKF than when estimated by MDC–KF. Both filters reduced the localisation error but (as observed along planned path 1), the localisation effect of the proposed method exceeded that of MDC–KF.

**Figure 12:**
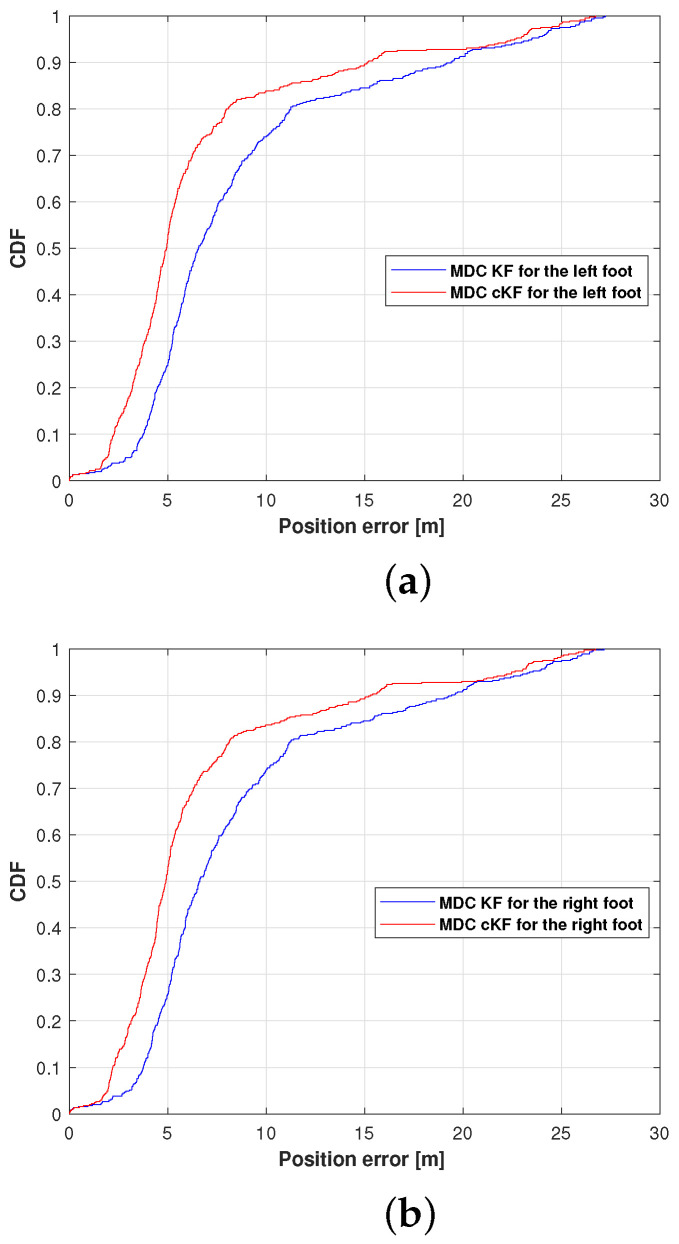
Position-error CDFs of the MDC–KF and MDC–cKF methods (solid blue and solid red lines, respectively) along planned path 2: (**a**) left foot, (**b**) right foot.

Figure 12 shows the CDFs of the position errors in the left and right feet measured by the MDC–KF and MDC–cKF methods along planned path 2. At the 0.9 CDF point, the proposed MDC–cKF obtained a smaller position error than the MDC–KF method, reconfirming the effectiveness of the proposed method.

**Figure 13:**
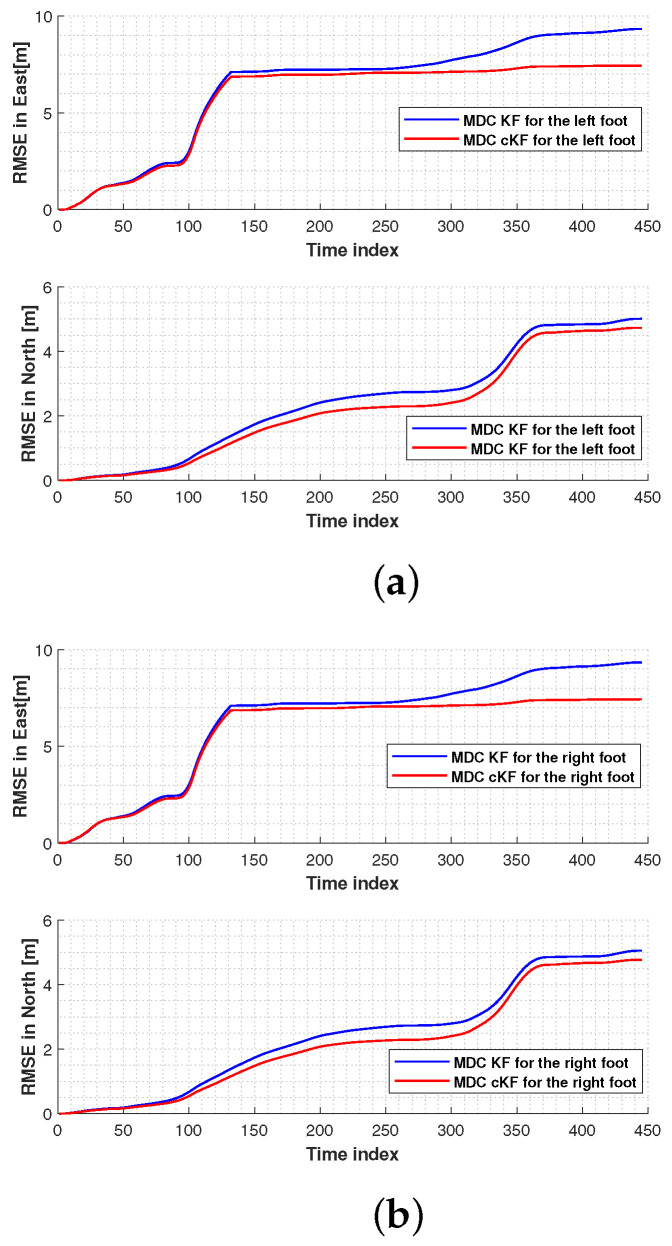
RMSEs in the foot positions measured by the MDC–KF and MDC–cKF methods (solid blue and solid red lines, respectively) along planned path 2: (**a**) left foot, (**b**) right foot.

Figure 13 shows the RMSEs in the foot measurements of the MDC–KF and MDC–cKF methods. In both the east and north directions, the proposed MDC–cKF method more effectively reduced the localisation error of the left foot than the MDC–KF method (Figure 13a). A similar result is observed for the right foot (Figure 13b).

To reconfirm the efficacy of the introduced methodology, we list the foot-position RMSEs of the MDC–KF and MDC–cKF methods along planned path 2 in Table 5 and Table 6, respectively. The MDC–KF method yielded precise mean position errors of 7.1724 m and 7.1935 m for the left and right foot, respectively. The proposed MDC–cKF method reduced these value to 6.0881 m and 6.1008 m, respectively, demonstrating improvements of 15.12% and 15.19%, respectively. The proposed method substantially improves the localisation accuracy from that of MDC–KF.

### 4.4. Localisation Performance Along Planned Path 3

This subsection evaluates and discusses the localisation performance of the proposed method along planned path 3 again employing MDC–KF as the benchmark method. Noted that in tests 1 and 2, the target person walked along planned paths 1 and 2, while in this experiment, the target person runs along the planned path 3.

**Figure 14:**
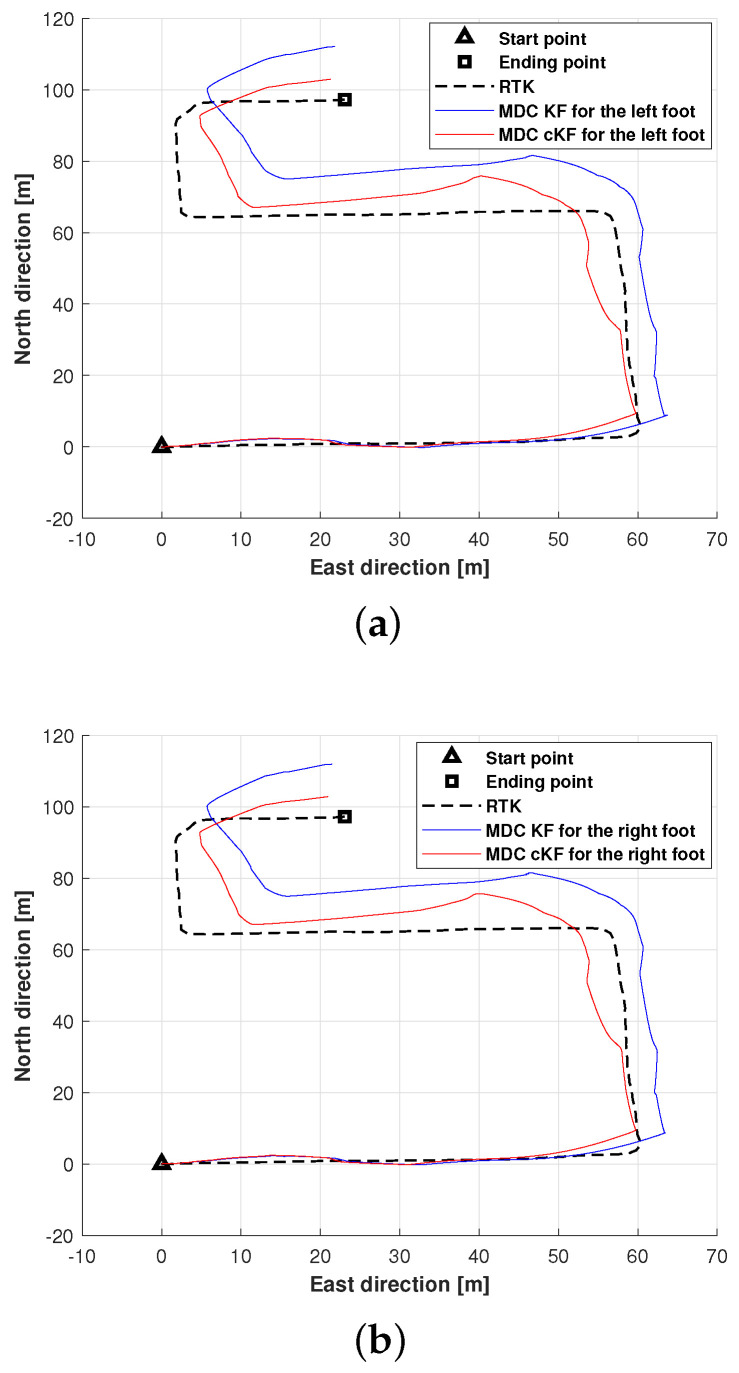
Foot positions along planned path 3 (black dashed lines) measured by the MDC–KF and MDC–cKF methods (solid blue and red lines, respectively): (**a**) left foot, (**b**) right foot.

The positions of the left and right feet measured by MDC–KF and MDC–cKF along planned path 3 are shown in Figure 14. Although the filters effectively reduced the localisation error, neither method prevented error accumulation. The positions of both feet more closely approached the planned path when estimated by MDC–cKF than when estimated by MDC–KF.

**Figure 15:**
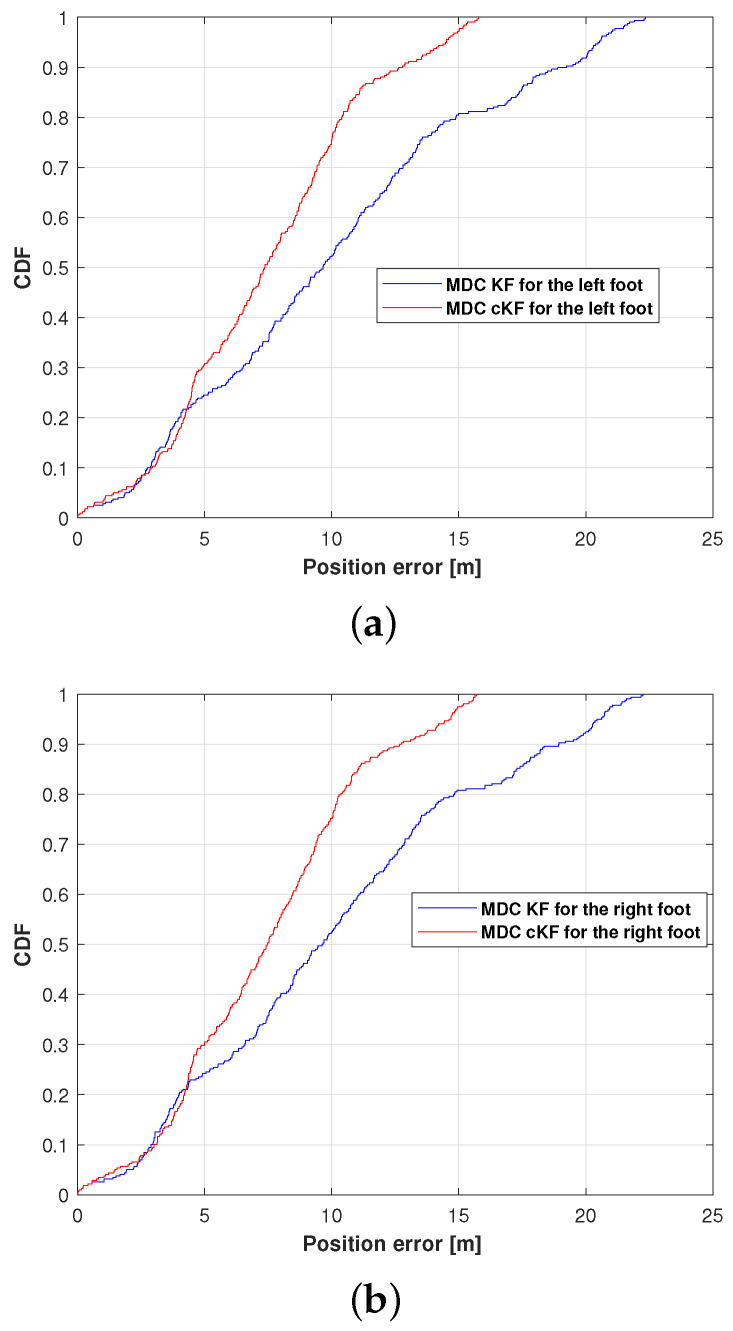
Position-error CDFs of the MDC–KF and MDC–cKF methods (solid blue and solid red lines, respectively) along planned path 3: (**a**) left foot, (**b**) right foot.

The CDFs of the position errors in the left and right feet measured by the MDC–KF and MDC–cKF methods along planned path 3 are shown in Figure 15. At the 0.9 CDF point, the proposed MDC–cKF obtained a smaller position error than the MDC–KF method, reconfirming the effectiveness of the proposed method even when the target person runs along the planned path 3.

**Figure 16:**
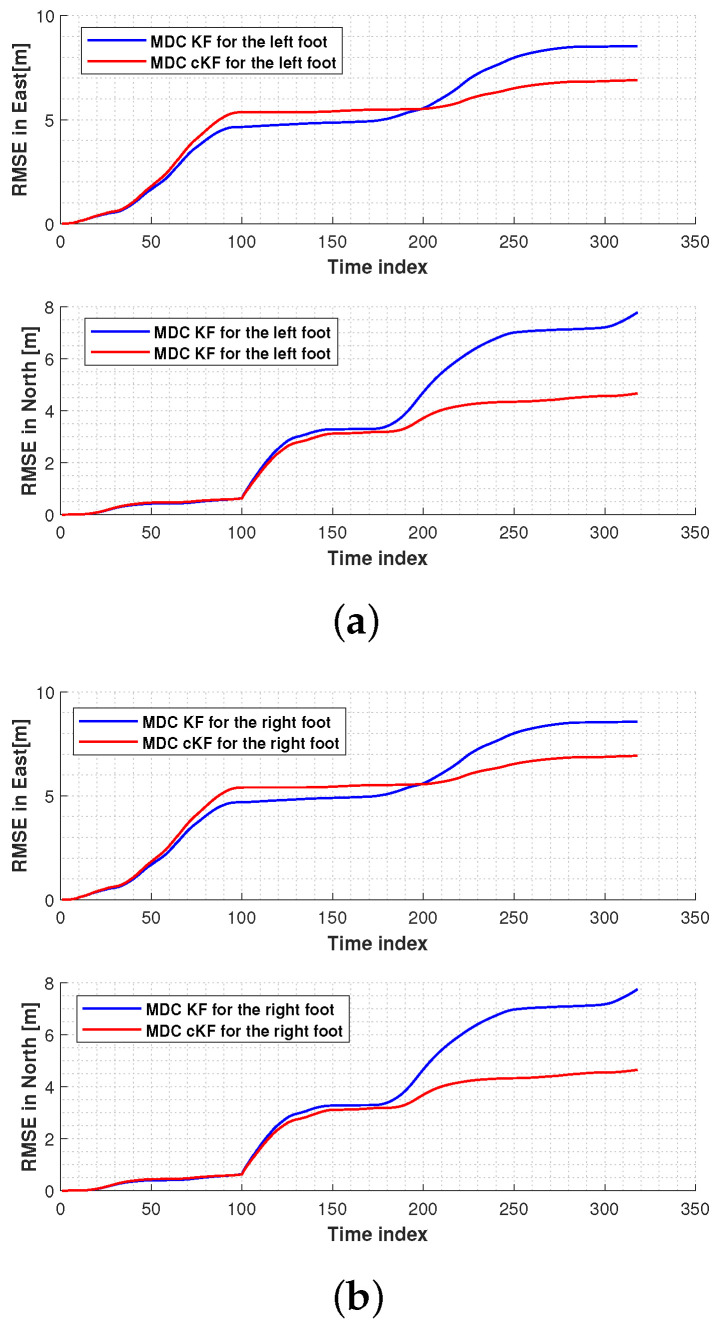
RMSEs in the foot positions measured by the MDC–KF and MDC–cKF methods (solid blue and solid red lines, respectively) along planned path 3: (**a**) left foot, (**b**) right foot.

The RMSEs in the foot measurements of the MDC–KF and MDC–cKF methods are shown in Figure 16. In both the east and north directions, the proposed MDC–cKF method more effectively reduced the localisation error of the left foot than the MDC–KF method (Figure 16a). A similar result is observed for the right foot (Figure 16b).

To reconfirm the efficacy of the introduced methodology, we list the foot-position RMSEs of the MDC–KF and MDC–cKF methods along planned path 3 in Table 7 and Table 8, respectively. The MDC–KF method yielded precise mean position errors of 8.1608 m and 8.1566 m for the left and right foot, respectively. The proposed MDC–cKF method reduced these value to 5.7815 m and 5.7869 m, respectively, demonstrating improvements of 29.15% and 29.05%, respectively. The proposed method substantially improves the localisation accuracy from that of MDC–KF.

### 4.5. Localisation Performance Along Planned Path 4

**Figure 17:**
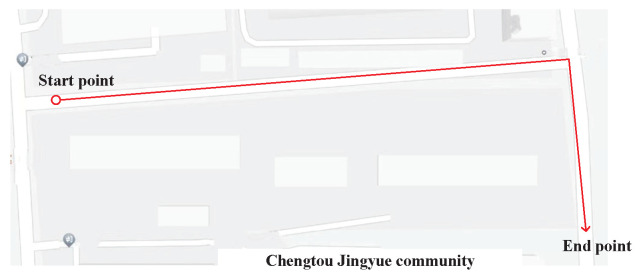
Planned path 4.

Moreover, we walks followed the trajectory shown in Figure 17 three times, and the results can be founded in Table 9 and Table 10. From the table, we can see that the mean position errors of the MDC–KF method for three times is 5.4901 m, and those value of the MDC–cKF method is 3.5969 m, which is improved by about 34.48% compared with the MDC–KF method.

## 5. Conclusions

This study investigated a human-localisation system using foot-mounted IMUs under CMN. We first designed a dual foot-mounted IMU-based human-localisation scheme in which an IMU is attached to each foot of the wearer. Both IMUs measure their acceleration, gyroscope, and magnetometer data in parallel. The data of each foot are then input to a data-fusion filter, which outputs the position. Based on the proposed localisation scheme, we derive a Kalman filter under CMN. The CMN is reduced by employing colour factors and modifying the traditional KF using the BE method. The modified KF is activated in the stance state of the human foot. Considering the CMN, we finally impose a constraint on the Kalman filter. As the constraint condition, we employ the minimum distance because a minimum distance between the feet on the plane was found during human walking. The constraint effectively reduces the INS’s position error of the left or right foot. Experimental results confirmed the superiority of MDC–cKF over MDC–KF. The proposed method improved the location accuracy and also reduced the runtime, affirming its high efficiency and practicability. Currently, we are introducing an interactive multiple model to improve the adaptability of the proposed method. We are also studying how the proposed method can enhance our understanding of processes that are influenced by coloured and correlated noise. We plan to report the results of these investigations in the near future.

## Figures and Tables

**Table 1:** The parameters of the IMUs used in this work.

Parameter	Value
Axes	3
Size	36.30×30.35×10.80mm
Dynamic (tilting heading)	$1.0^{\circ }\;|2.0^{\circ }1\sigma \;\mathrm{RMS}$
Stance (tilting heading)	$0.5^{\circ }\;|1.0^{\circ }1\sigma \;\mathrm{RM}S$
Temperature range	0 °C∼50 °C

**Table 2:** The parameters of the R-T-K sensor used in this work, which has been used in [3,28], and the present work.

Parameter	Value
R-T-K accuracy (plane)	$\pm \left(8+1\times 10^{6}\times D\right)\mathrm{mm}$
R-T-K accuracy (height)	$\pm \left(15+1\times 10^{6}\times D\right)\mathrm{mm}$
Size	119mm×119mm×85mm
Weigh	0.73 kg
Operation temperature	−45 °C∼+75 °C

**Table 3:** RMSEs in the left-foot positions measured by MDC–KF and MDC–cKF along planned path 1.

Methods	East Position	North Position	Mean
MDC–KF	13.8807	5.7767	9.8287
MDC–cKF	7.8685	4.4977	6.1831

**Table 4:** RMSEs in the right-foot positions measured by MDC–KF and MDC–cKF along planned path 1.

Methods	East Position	North Position	Mean
MDC–KF	13.8432	5.82	9.8316
MDC–cKF	7.8712	4.5123	6.1918

**Table 5:** RMSEs in the left-foot positions measured by MDC–KF and MDC–cKF along planned path 2.

Methods	East Position	North Position	Mean
MDC–KF	9.3362	5.0087	7.1724
MDC–cKF	7.4472	4.729	6.0881

**Table 6:** RMSEs in the right-foot positions measured by MDC–KF and MDC–cKF along planned path 2.

Methods	East Position	North Position	Mean
MDC–KF	9.3328	5.0542	7.1935
MDC–cKF	7.4313	4.7704	6.1008

**Table 7:** RMSEs in the left-foot positions measured by MDC–KF and MDC–cKF along planned path 3.

Methods	East Position	North Position	Mean
MDC–KF	8.5421	7.7795	8.1608
MDC–cKF	6.8992	4.6638	5.7815

**Table 8:** RMSEs in the right-foot positions measured by MDC–KF and MDC–cKF along planned path 3.

Methods	East Position	North Position	Mean
MDC–KF	8.5637	7.7496	8.1566
MDC–cKF	6.9207	4.6531	5.7869

**Table 9:** RMSEs in the right-foot positions measured by MDC–KF along planned path 4.

Times	East Position	North Position	Mean
1	10.37	2.7585	6.5642
2	9.3585	1.796	5.5772
3	5.6617	2.8161	4.2389

**Table 10:** RMSEs in the right-foot positions measured by MDC–cKF along planned path 4.

Times	East Position	North Position	Mean
1	4.8844	2.0522	3.4683
2	6.0716	1.7067	3.8891
3	4.2639	2.6029	3.4334

## Data Availability

The original contributions presented in the study are included in the article, further inquiries can be directed to the corresponding author.

## Abbreviations

The following abbreviations are used in this manuscript:

BE	Backward Euler
CDF	Cumulative distribution function
CMN	Coloured measurement noise
EKF	Extended Kalman filter
GPS	Global positioning system
GNSS	Global navigation satellite system
KF	Kalman filter
IMU	Inertial measurement unit
IMM	Interacting multiple model
INS	Inertial navigation system
RFID	Radio frequency identification
R-T-K	Real-time-kinematic
RMSE	Root mean square error
PDR	Pedestrian dead reckoning
MDC	Minimum-distance-constraint
